# Scalable Parallel Manipulation of Single Cells Using Micronozzle Array Integrated with Bidirectional Electrokinetic Pumps

**DOI:** 10.3390/mi11040442

**Published:** 2020-04-22

**Authors:** Moeto Nagai, Keita Kato, Satoshi Soga, Tuhin Subhra Santra, Takayuki Shibata

**Affiliations:** 1 Department of Mechanical Engineering, Toyohashi University of Technology, Toyohashi, Aichi 441-8580, Japan; bari91_ape50@yahoo.co.jp (K.K.); ss3104u5@gmail.com (S.S.); shibata@me.tut.ac.jp (T.S.); 2 Department of Engineering Design, Indian Institute of Technology Madras, Tamil Nadu 600036, India; santra.tuhin@gmail.com

**Keywords:** micronozzle-array, parallel cell manipulation, bidirectional electrokinetic pump, DC biased AC electrokinetic flow

## Abstract

High throughput reconstruction of in vivo cellular environments allows for efficient investigation of cellular functions. If one-side-open multi-channel microdevices are integrated with micropumps, the devices will achieve higher throughput in the manipulation of single cells while maintaining flexibility and open accessibility. This paper reports on the integration of a polydimethylsiloxane (PDMS) micronozzle array and bidirectional electrokinetic pumps driven by DC-biased AC voltages. Pt/Ti and indium tin oxide (ITO) electrodes were used to study the effect of DC bias and peak-to-peak voltage and electrodes in a low conductivity isotonic solution. The flow was bidirectionally controlled by changing the DC bias. A pump integrated with a micronozzle array was used to transport single HeLa cells into nozzle holes. The application of DC-biased AC voltage (100 kHz, 10 V_pp_, and V_DC_: −4 V) provided a sufficient electroosmotic flow outside the nozzle array. This integration method of nozzle and pumps is anticipated to be a standard integration method. The operating conditions of DC-biased AC electrokinetic pumps in a biological buffer was clarified and found useful for cell manipulation.

## 1. Introduction

Functional analysis of cells leads to new insights in the medical and pharmaceutical fields. Mimicking in vivo cellular environments in vitro is key to efficient analysis. In organs, cells communicate with each other via direct contact over short or long distances and are influenced by the cellular microenvironment, e.g., cell–cell and cell–matrix interactions. Heterotypic cell–cell interactions have been studied using in vitro co-culture systems [1,2,3]. Microfabrication and microfluidic technologies were used for co-cultures of cells and cell-cell interactions were studied [4,5]. However, conventional in vitro techniques are often insufficient for reconstructing cellular microenvironments in any combination. Single cell manipulation tools [6] are required for mimicking the in vivo environment in vitro with a higher reproducibility.

Single cells have been manipulated by fluidic [7,8,9,10,11], dielectrophoretic [12,13,14], and optical [15,16,17] techniques and have been encapsulated in droplets [18,19]. Previous methods had limited accessibility and did not offer sufficient versatility and flexibility. Accessibility to an open-top chip and high flexibility are provided by one-side-open devices such as a glass capillary [20,21], a cantilever with an aperture [22,23], dielectrophoretic tweezers [24,25], inkjet-like printing [26,27], and printed droplet microfluidics [28]. These devices are typically composed of a single probe, and their throughput is limited because of the limit on the number of channels. Increasing the number of channels enables higher throughput in manipulation of single cells while maintaining flexibility and open accessibility. The number of micro-channels for dispensing droplets containing single cells was increased to three, and three pneumatic pressure sources and solenoid valves were integrated into the system [29]. The manipulation of single cells was demonstrated with a hollow probe array and a single pressure source in open space [30], even though individual flow control of each micro-channel remained an issue.

Mechanical and electrical pumps [31] can be integrated with one-side-open multi-channel devices for individual flow control. Among these pumps, electroosmotic pumps (EOPs) [32] are easy to integrate with microdevices and scalable. DCEOPs have the advantage of simplicity [33,34] and bidirectional flow, but have a potential issue with bubble formation due to electrolysis. To avoid electrolytic reactions, ACEOPs have been developed. Although ACEOPs can change flow direction using a travelling wave [35,36], this method requires phase shift and is relatively difficult to scale. A DC-biased AC-electrokinetic (ACEK) flow was used to induce bidirectional flow [37]. W.Y. Ng et al. investigated the mechanism of the flow and concluded that a conductivity gradient drives fluid flow [38,39]. Deionized (DI) water [37,40], a 10^−4^ M solution of KCl (conductivity 1 mS m^−1^), and a CuSO_4_ solution (conductivity 4 mS m^−1^) [38] were used as working fluids. However, the availability of an isotonic solution for DC-biased ACEKPs is unclear.

A method of integrating polydimethylsiloxane (PDMS) micro-nozzles, micro-channels, and pump electrodes for parallel manipulation of single cells was developed in this study. Interdigital tooth electrodes were made from indium tin oxide (ITO) or Pt/Ti, and a DC-biased AC signal applied to them. We characterized electrokinetic flow in an isotonic solution at both electrodes. The DC bias voltage was switched to control the direction of flow. Single cells were manipulated with a micro-nozzle array integrated with DC-biased ACEKPs. We characterized the manipulation of single cells with the nozzles.

## 2. Experimental Materials and Methods 

### 2.1. Concept and Design of PDMS Nozzle Array Integrated with Electrokinetic Micropumps

**Figure 1:**
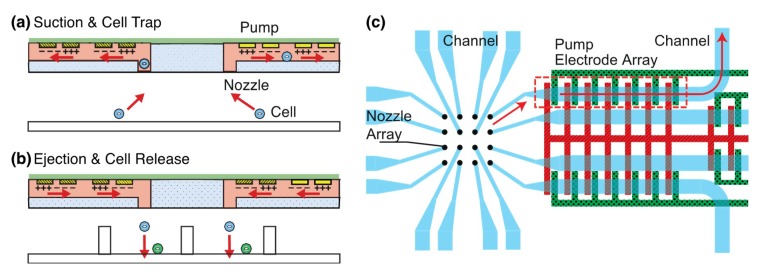
The schematic of a nozzle array integrated with electrokinetic pumps for cell manipulation. (**a**) Suction and trap of single cells in an array format with nozzles. (**b**) Ejection and release of the cells from the nozzles. (**c**) Schematic view of 4 × 4 nozzles integrated with pumps. One of the electrodes (green) is connected to a signal voltage (DC-biased AC voltage) and the other electrode (red) is connected to the ground.

The integration of DC-biased ACEK pumps into nozzles enables them to transport fluid in both directions (Figure 1). Since symmetric electrodes were used, the positive and negative of the DC bias were changed for bidirectional flow control. The diameter of micronozzles was designed to be around 30 μm for manipulating multiple types of single cells approximately 10–20 μm in diameter simultaneously. Cells are sucked into microchannels by the actuation of pumps (Figure 1a). The cells are trapped, moved to the top of microwells, and ejected from the channel to make a pair of cells (Figure 1b). An array of 4 × 4 micronozzles is integrated with DC-biased ACEKPs (Figure 1c), which are composed of seven pairs of electrodes. 

The principles of the pump is based on DC-biased AC-electrokinetics [38]. Planar parallel electrodes are placed in a microfluidic channel in contact with an electrolyte solution and a DC biased AC electrical signal is applied to the electrode pair. The application of DC bias induces Faradaic electrolytic reactions and results in an increase of the ionic content of the bulk solution. The ionic contents differ at the cathodic and anodic sides. DC biased AC electric signal acts on the transverse conductivity gradient and generates fluid flow.

An array of 4 × 4 nozzles was arranged symmetrically with a pitch of 200 µm and concentrated in the middle region (680 µm × 680 µm) for microscopic observation of as many cells as possible. Each 60-µm width channel was connected to a 30-µm diameter nozzle for the concentration and the minimum spacing between channels was set at 30 µm. We followed the dimensions of electrodes for bidirectional flow transport from a previous study [37], where they were 80-µm wide with gaps of 20 µm and 100 µm. A lower-height channel induces higher flow velocity and we selected 70 µm as the channel height.

### 2.2. Fabrication of PDMS Nozzle Array Integrated with Electrokinetic Micropumps

**Figure 2:**
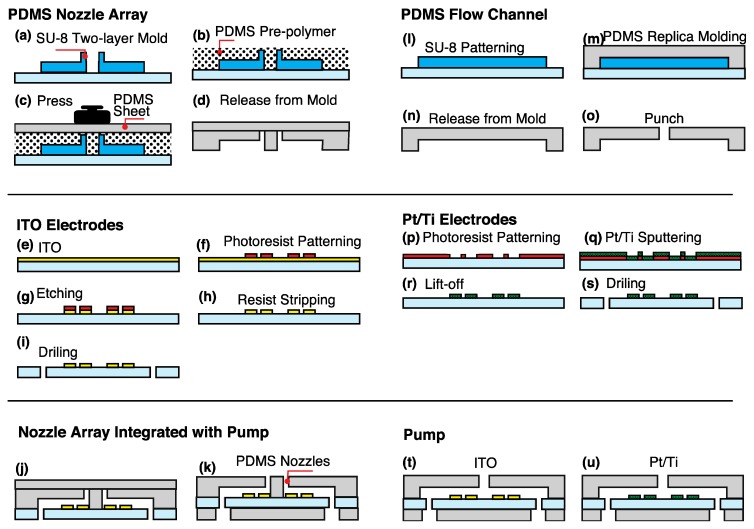
Fabrication process of the nozzle array integrated with pump. (**a**–**d**) Fabrication of polydimethylsiloxane (PDMS) nozzles. Uncured PDMS was pressed by a PDMS sheet to form PDMS through holes. (**e**–**i**) Patterning of OFPR photoresist on indium tin oxide (ITO) substrate and formation of ITO electrodes. ITO was etched with hydrochloric acid. (**j**,**k**) Bonding of the PDMS micronozzles, ITO electrodes, and a PDMS sheet after plasma treatment. (**l**–**o**) Fabrication of PDMS flow channel. (**p**–**s**) Patterning of Pt/Ti electrodes. (**t**–**u**) Bonding of the PDMS microchannel, ITO, or Pt/Ti electrodes, and a PDMS sheet after plasma treatment.

Then, 4 × 4 PDMS nozzles were integrated with electrokinetic ITO pumps for single cell manipulation. PDMS micronozzles were formed and combined with an ITO substrate patterned on a glass. First, a method of making PDMS though holes [41,42] for fabricating PDMS nozzles was used and the method extended from a single-layer mold to a double-layer mold. Double-layer thick photoresist, SU-8 3050 (Kayaku Microchem, Tokyo, Japan), was formed on a 3-inch Si substrate (Figure 2a). A silicon wafer was dehydrated at 160 °C for 5 min. The first layer of SU-8 was spincoated at 500 rpm for 25 s and 1000 rpm for 55 s to obtain a film thickness of 70 μm. It was pre-baked at 60 °C for 5 min, at 95 °C for 50 min, and at 60 °C for 5 min. The pattern was exposed through a PMMA filter and mask aligner (PEM-800, Union Optical Co., Ltd., Tokyo, Japan). The integrated exposure light was 4675 mJ/cm^2^ (contact mode). The wafer was post-baked at 65 °C for 9 min, at 95 °C for 5 min, and at 65 °C for 2 min. The second SU-8 layer was spin-coated at 500 rpm for 25 s and 1000 rpm for 55 s to make the micronozzles after PDMS molding. The wafer was prebaked at 60 °C for 5 min, at 95 °C for 50 min, and at 60 °C for 5 min. The SU-8 was exposed to the integrated exposure light (4675 mJ/cm^2^) through a mask and post-baked at 65 °C for 9 min, 95 °C for 5 min, and 65 °C for 2 min. The SU-8 molds of the microchannel or micronozzles were developed in 2-acetoxy-1-methoxypropane (Wako Pure Chemical Industries Ltd., Osaka, Japan) for 15 min. They were rinsed with isopropyl alcohol. The SU-8 molds were treated with vapor of trichloro(1H,1H,2H,2H-perfluorooctyl)silane (PFOCTS).

PDMS (Silpot 184, Dow Corning Toray Co., Ltd., Tokyo, Japan) was mixed at a 10:1 ratio of the base polymer to curing agent. Uncured PDMS was poured over the fabricated SU-8 molds (Figure 2b). A 3-inch diameter and 3-mm thick PDMS sheet was formed as the carrier sheet and treated with PFOCTS. Uncured PDMS was pressed with the PDMS carrier sheet and a weight of about 600 g (Figure 2c). PDMS was cured at room temperature for 24 h to expel PDMS from SU-8 pillars. The PDMS were released from the mold through holes on the sheet (Figure 2d). 

A 200-nm thick ITO-coated 100 mm × 100 mm glass substrate (thickness 0.7 mm) was purchased from Geomatec Co., Ltd. (Yokohama, Japan) and used as the electrode (Figure 2e). A substrate was cut into a33 mm × 33 mm piece and ultrasonically cleaned for 5 min with acetone, isopropyl alcohol (IPA), and DI water each. An ITO substrate was dehydrated at 160 °C for 5 min. Hexamethyldisilazane (HMDS) and OFPR-8600 (52 cp, Tokyo-Oka Kogyo Co., Ltd., Kawasaki, Japan) were spin-coated on the substrate at 1000 rpm for 5 s and 2000 rpm for 25 s. The thickness of OFPR was increased to resist etching in hydrochloric acid. The substrate was prebaked at 110 °C for 90 s. OFPR was exposed to an integrated light quantity of 100 mJ/cm^2^ to form pump electrodes. The resistance was immersed in a developer (NMD-3, Wako Pure Chemical Industries, Ltd.) for 120 s, rinsed with DI water for 2 min, and blown with N_2_ gas (Figure 2g). The resistance was post-baked at 140 °C for 5 min. ITO was etched in 9 mol/L hydrochloric acid at 40 °C for 6 min using OFPR as a mask (Figure 2h). The resistance on the bare ITO was checked to fully etch ITO. The resistance was removed by ultrasonic cleaning with acetone for 5 min. A 1-mm hole was drilled in the glass part of the ITO substrate for liquid introduction (Figure 2i).

The PDMS chip and ITO electrodes were treated with air plasma (plasma treater, YHS-R, Sakigake Semiconductor Co., Ltd., Kyoto, Japan) for 60 s for bonding. The substrates were aligned with mechanical stages and permanently bonded by baking them at 80 °C for 40 min (Figure 2j). The cured PDMS was peeled off from the PFOCTS-treated PDMS sheet. A PDMS sheet with 0.5-mm holes was bonded on the back side to introduce into the solution after plasma treatment. The micronozzles were integrated with the pumps (Figure 2k).

### 2.3. Fabrication of EOF Pump from ITO and Pt/Ti Electrodes

Interdigital electrodes were patterned on a glass substrate (Figure 2e–i,p–s) and bonded to a PDMS flow channel. A PDMS channel was molded from the SU-8 structure. The electrode was an interdigital structure in which seven symmetrical electrodes were arranged at regular intervals. ITO and Pt/Ti were used as the electrode material. For the Pt/Ti electrode, Ti was deposited as an adhesion layer of Pt and glass, and Pt was utilized as the electrode surface.

The PDMS flow channel was prepared as in Section 2.1. A single-layer thick photoresist, SU-8 3050, was used for a mold of the PDMS micro-channel (Figure 2l). PDMS was cured at 80 °C and microchannels were molded in PDMS from the SU-8 mold (Figure 2m) and released (Figure 2n). A 0.5 mm-hole was punched at the end of the PDMS flow channel to introduce and eject a solution (Figure 2o).

A slide glass (76 × 26 × 1 mm, No. 1, Matsunami Glass Industry Co., Ltd., Osaka, Japan) was cut into three equal parts (about 26 mm × 26 mm) for the Pt/Ti electrodes. The glass was cleaned in a mixture of sulfuric acid and hydrogen peroxide mixture (SPM) and dehydrated at 140 °C (5 min). HMDS and OFPR-8600 were spin-coated at 3000 rpm for 20 s. The glass was pre-baked at 110 °C for 90 s. The resist was exposed to the integral amount of 250 mJ/cm^2^ (contact mode) and developed in NMD-3 for 10 min (Figure 2p). The glass was rinsed with DI water for 2 min and post-baked at 140 °C for 5 min. A Ti film (10-nm thick film) and Pt film (100-nm thick film) were deposited on three glass slides for 1 min as an adhesion layer by RF magnetron sputtering (L-250S-FH, Anelva Co., Ltd., Tokyo, Japan) (Figure 2q). Pt/Ti liftoff was performed in acetone with an ultrasonic cleaner (100 kHz) for 20 min (Figure 2r). The surface was then cleaned with IPA and nitrogen gas was blown off. The glass was drilled with a 1-mm diameter to form a hole for liquid introduction (Figure 2s). The ITO or Pt/Ti electrodes and PDMS micro-channels were bonded after air plasma (Figure 2t,u).

### 2.4. Observation Setup and Voltage Application

An inverted microscope (ECLIPSE Ti-U, Nikon Co., Ltd., Tokyo, Japan), equipped with ×4, ×10, and ×20 objective lenses, and a cooled charge-coupled device (CCD) camera (DS-Qi1Mc, Nikon) were used for flow observation. The movement of particles or cells transported with electrokinetic micropumps at 13–15 fps was recorded. 

Voltage was applied to the interdigital electrodes by connecting it to a function generator (Protek 9305, GS Instruments Co., Ltd., Incheon, Korea) via an Ag/AgCl electrode. One electrode was set to ground (GND) and the other electrode was set to a signal electrode. We applied AC voltage with a DC bias voltage V_DC_ in the range of +4 V to −4 V at a frequency of 100 kHz and a peak value V_pp_ of 8 V to 12 V.

### 2.5. Flow Characterization of Electrokinetic Flow Pump

The DC-biased ACEK flow was characterized as having ITO or Pt/Ti electrodes and a low conductivity isotonic solution buffer (8.5 w/v% sucrose and 0.3 w/v% glucose) [43] was used to suppress the influence of Joule heating in the solution. Fluorescent particles (Sigma, L4530, particle diameter 2.0 μm, concentration 2.86 × 10^7^ particles/mL) were suspended in the low conductivity buffer and were used as a tracer. The solution was introduced into the flow channel with a syringe. After the introduction, the hole was covered with a glass coverslip to reduce the influence of flow generated by a pressure imbalance.

The particles were transported in the flow channel of the electrokinetic pump to evaluate their driving characteristics. We traced 10 particles from 10 s after from the start of voltage application and measured their moving distance for 30 frames (about 2.2 s). The average particle velocity was derived from the movement of the particles and was considered as flow velocity. The change in the electrokinetic flow velocity was investigated by changing voltage conditions.

### 2.6. Manipulation of Cells Using a Micro-Nozzle Array Integrated with an Electrokinetic Flow Pump 

**Figure 3:**
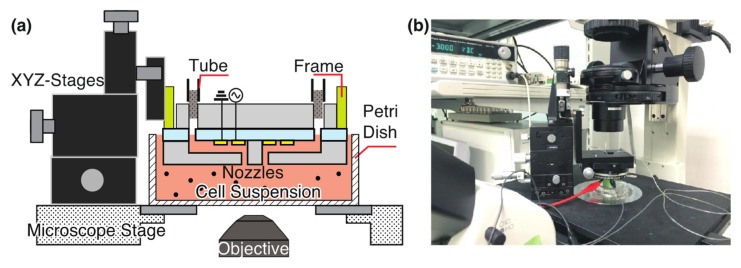
The experimental setup for cell manipulation with a developed nozzle array. (**a**) Schematic and (**b**) picture of the setup. A nozzle array was mounted on the XYZ stage via a 15 mm × 15 mm stainless frame. Electrodes were connected with AC power sources and ground (GND). In the channel of a nozzle array, particle suspension was introduced and HeLa cells were suspended in a petri dish.

We generated a DC-biased ACEK flow and manipulated cells through the fabricated micro-nozzle array. An overview of the experiment setup is shown in Figure 3. The micro-nozzle array was fixed to XYZ stages via a frame. We adjusted the position on the XYZ stage so that the nozzle surface was close to the bottom of the Petri dish. The nozzle array was placed in a cell suspension. The electrodes were connected to the function generator and GND. HeLa cells (RIKEN, RCB0007 provided by the RIKEN BioResource Research Center through the National BioResource Project of the MEXT/AMED, Tsukuba, Japan), were suspended in a low conductivity buffer containing φ2.0 μm tracer particles. The cell concentration was adjusted to 4.28 × 10^5^ cells/mL and the cell suspension was poured in a petri dish. The nozzle array was brought close to the bottom of the petri dish. A DC-biased AC signal was applied to the electrodes connected to one nozzle to generate an electrokinetic flow, and cells through single nozzles were manipulated. The applied signal was V_DC_ −4 V, frequency of 100 kHz, and V_pp_ 10 V.

## 3. Results and Discussion

### 3.1. Fabrication of the Integrated Nozzle Array and Pump

**Figure 4:**
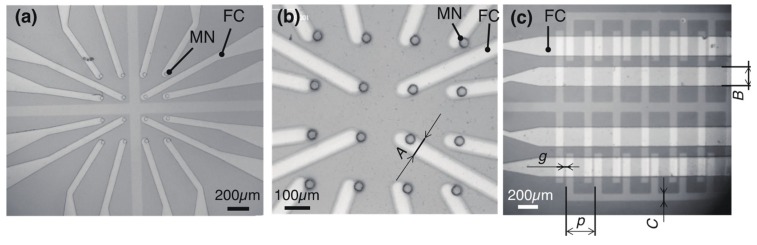
Micrographs of micro-nozzles integrated with ITO micropumps. (**a**) 4 × 4 micro-nozzles connected with micro-channels. Nozzle and channel were fabricated in the transparent materials PDMS and glass, which enabled observation of any transmission. (**b**) Close-up view of a micro-nozzle array. (**c**) Four series of flow channels and electrodes. FC and MN denote flow channel and a micro-nozzle, respectively. Typical dimensions were measured from five points: Nozzle diameter 34.8 μm. Channel width, *A* = 62.0 μm. *B* = 189.1 μm. Height, *H* = 71.9 μm. Electrode width, *C* = 71.9 μm. Gap *g* = 27.8 μm. Pitch *p* = 278.8 μm.

An array of 4 × 4 micronozzles integrated with electrokinetic flow pumps was fabricated (Figure 4, Table 1). Each part was fabricated by molding, lift-off, and etching. The nozzle array integrated with pumps were made of transparent PDMS and ITO electrodes, which enabled observation of the inside of a channel. Additionally, 4 × 4 nozzles were opened as water passed through the nozzles. The nozzle’s inner diameter was 34.8 ± 1.04 μm (N = 16, mean ± standard deviation) (Figure 4a,b) and enlarged from 30 µm. PDMS micro-nozzles were connected to flow channels and ITO electrodes were patterned on a glass substrate (Figure 4c). The electrokinetic flow pump consisted of seven pairs of electrodes. The ITO interdigital electrodes were arranged in the flow channel with a width of 71.9 μm, a gap of 27.8 μm, and a pitch of 278.8 μm. The flow channel and pump were separated from each other by the PDMS wall, and each pump had its own signal input electrode. This design enabled individual flow control. The advantage of employing electrical pumps for cell transport was that it provided true scalability to one-side-open multi-channel devices. 

ITO or Pt/Ti micropumps with single-layer micro-channels and a 0.5-mm hole for characterization of DC-biased ACEKPs were also fabricated. Both the electrodes were arranged at regular intervals.

### 3.2. Characterization of DC-Biased ACEK Flow Using Electrokinetic Flow Pump

**Figure 5:**
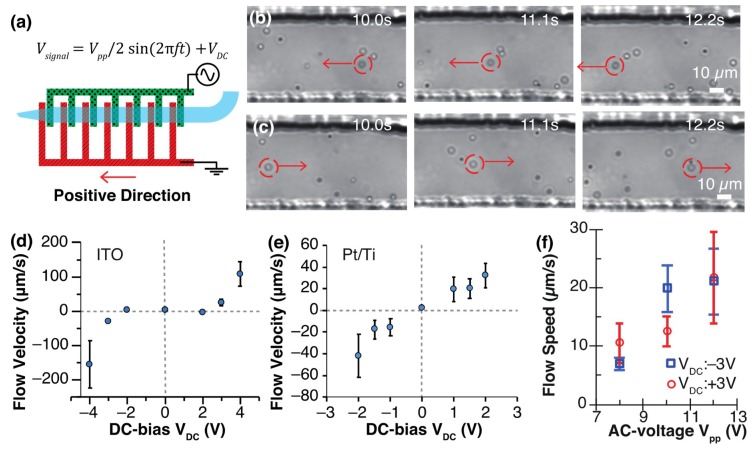
Generation of DC-biased AC electrokinetic flow with an ITO or Pt/Ti pump. The frequency and peak-to-peak voltage of the AC signal were set to 100 kHz and V_pp_ 10 V, respectively. (a) Schematic diagram of the pump. Particle transportation with an ITO pump at (**b**) V_DC_ +3 V and (**c**) V_DC_ –3 V. Electrokinetic flow velocity versus DC bias with (**d**) ITO and (**e**) Pt/Ti electrodes. (**f**) Flow speed versus V_pp_ voltage with ITO electrodes. Flow velocity was measured from 10 particles moving in the channel.

DC bias was switched to positive or negative for generation of a bidirectional flow. Figure 5 shows the transport of particles (φ2 μm) in a low conductive isotonic buffer at V_pp_ 10 V, 100 kHz, and V_DC_ ± 3 V. Unless stated, V_pp_ 10 V and 100 kHz were used. The application of positive and negative V_DC_ generated a net flow from the signal electrode to the ground electrode and vice versa. The voltage application of V_DC_ +3 V and −3 V moved particles from the electrode side to the nozzle side (Figure 5b, Movie S1) and from the nozzle side to the electrode side (Figure 5c, Movie S2). The application of voltage immediately created flow and the shut-down of voltage application stopped the generated flow. Movie S3 presents a vortex flow near a pair of ITO electrodes, and the creation of a net bidirectional flow through a PDMS micro-channel. 

The average flow velocity was obtained according to the bias voltage (V_DC_), where the direction from the signal electrode to the ground electrode was defined as positive (Figure 5d–f). The flow speed increased with an increase in the applied DC bias at both the ITO electrode and the Pt/Ti electrode (Figure 5d,e). With the ITO pump, the flow velocities were positive at V_DC_ +3 V or greater, and negative at V_DC_ −3 V or less. The flow velocities were 108.7 ± 36.4 μm/s (N = 10) at V_DC_ + 4 V and −155.6 ± 69.5 μm/s (N = 10) at V_DC_ – 4 V. We calculated the maximum flow rate of the pump with the product of a flow speed of 156 μm/s and a cross-section area of 4.46 × 10^3^ μm^2^ (channel width of 62.0 μm × channel height of 71.9 μm). The flow rate of the pump was 6.96 × 10^5^ μm^3^/s, which was equal to 696 pL/s. The flow velocities are almost symmetrical about the origin, but there is some difference. A two-electrode system was employed for flow generation because of its simplicity, while the potential of the electrodes was difficult to control. The addition of reference electrodes and a three-electrode system would stabilize the potential of the electrodes and flow generation. Bubbles were generated between Pt/Ti electrodes at V_DC_ ±3 V and bridged a pair of the electrodes after 30 s of voltage application. Therefore, the flow velocities at V_DC_ = ±3 V and ±4 V were not measured. The comparison of electrode materials shows that ITO pumps generate a faster flow than Pt/Ti electrodes and are suitable for large-volume fluid pumping. Pt/Ti electrodes are more stable than ITO and satisfy a long period of usage.

While most of the cell dimensions are between 10–20 µm, we prioritized the measurement of the fluid velocity and chose φ2-µm tracer particles in our characterization experiments. The smaller particles decreased the inertia of tracers and the measurement error [44]. The smaller size particles improve the response to flow, whereas the response of HeLa cells is slower than the particles. The suction and release of cells are assumed to take more time than the results characterized with φ2-µm particles. The particle concentration was 2.86 × 10 particles/nL and this concentration was low enough to not cause interactions between particles.

ITO pumps did not generate a significant electrokinetic flow in the range of V_DC_ of −2 V to +2 V. The Pt/Ti pumps generated a significant electrokinetic flow at V_DC_ of ±1 V. While a bubble formed between a pair of Pt/Ti electrodes at V_DC_ of ±3 V, bubbles did not form between a pair of ITO electrodes at V_DC_ of ±3 V. Bubble generation relates to the electrolysis of the solution. The reason why ITO pumps did not create a clear electrokinetic flow with a small DC bias is because the voltage did not go above the overpotential of water electrolysis. The working principle of the DC-biased AC pump was assumed to be based on a transverse conductivity gradient. This gradient was created through incipient Faradaic reactions occurring at the electrodes when a DC-bias AC voltage was applied to gold electrodes [38]. The electrolytic decomposition reaction of water occurred, as shown below. H^+^ ions were generated at the anode side electrode.

2H_2_O(L) → 4H^+^ (aq) + O_2_(g) + 4e^−^ (1)

OH^−^ ions are generated from the cathode side electrode.

4H_2_O(L) + 4e^−^ → 4OH^−^ (aq) + 2H_2_(g) (2)

Because the difference in conductivity between H^+^ and OH^−^ was made in these reactions, a conductivity gradient was created in the solution on the anode and the cathode. This conductivity gradient and voltage application generated vortex flow between symmetrical electrodes. The change of electrode materials affected the overpotential of water electrolysis.

Peak-to-peak amplitude, *V_pp_* on speed of DC-biased ACEK flow were investigated. With increasing *V_pp_*, flow speeds increased (Figure 5f). Absolute flow speeds were plotted for ease of comparison. Flow speed was around 10 µm/s at V_pp_ 9 V and around 20 µm/s at V_pp_ 11 V for positive and negative DC bias. Based on the above observations, it was concluded that the larger the absolute value of the DC bias voltage or AC voltage, the greater the flow speed.

### 3.3. Cell Manipulation by Pump-Integrated Micro-Nozzle Array

**Figure 6:**
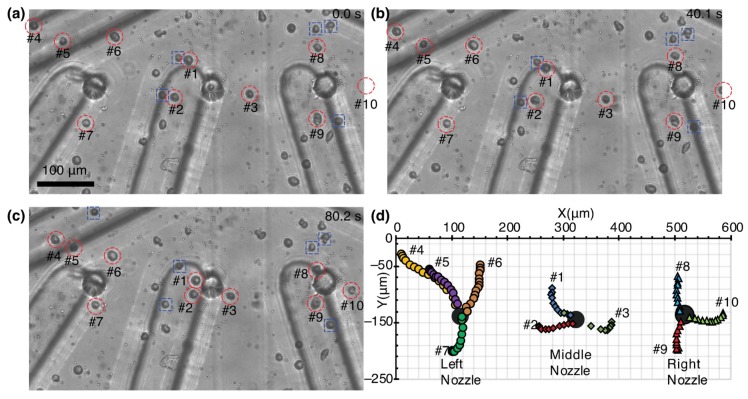
Suction of 10 cells using an electrokinetic pump near three nozzles (100 kHz, 10 V_pp_, V_DC_ −4 V). (**a**–**c**) Time-lapse images of cell suction. Voltage was applied at 0 s and the flow was continuously generated. The 10 cells are circled by a red dashed line and attracted cells are boxed by a blue dashed line. (**d**) Trajectories of the aspirated 10 cells.

We manipulated 10 cells in a parallel manner by electrokinetic flow with three of 16 nozzles (Figure 6, Movie S4). HeLa cells were suspended in the low conductive isotonic buffer and poured in the bottom of a Petri dish. A nozzle array was immersed in the cell suspension with mechanical stages. Before suction, cells distributed everywhere in the field. Since the largest flow velocity was obtained with ITO electrodes at V_DC_ ±4 V from the previous section, DC-biased AC voltages (V_DC_ −4 V, V_pp_ 10 V, 100 kHz) was applied and the pump generated an inward flow to the flow channel. During the application of the voltage, a suction flow was continuously created and vortex flow was generated in between a pair of electrodes. We focused on three nozzles for characterization rather than 16 nozzles due to the limitation of the observation area. When the gap between the nozzle array and the bottom of the dish was about 50 µm, only φ2-µm particles were sucked and cells were not sucked into the nozzles for 120 s. The gap was further reduced to around 20–30 µm and cells were transported. A cell denoted as #2 in Figure 6 was aspirated into the middle nozzle at about 76.6 s after the start of the application (Figure 6c). Each cell was attracted to the nearest nozzle (Figure 6d). These results indicate that the liquid volume should be reduced to create a sufficient flow for cell transportation. The nozzle array was fabricated in transparent materials such as PDMS, glass, and ITO, so that the structure did not prevent observation of the cells. 

**Figure 7:**
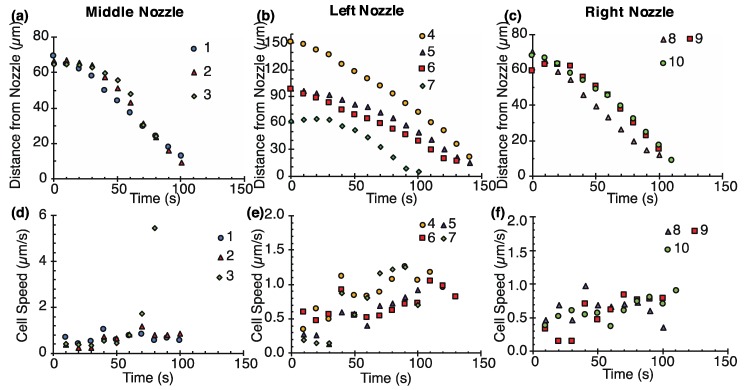
Characterization of parallel single cell suction. (**a**–**c**) Relationship between the distance of single cells from the nearest nozzle and time. (**d**–**f**) Relationship between the speed of the cells and time.

Ten cells near the nozzles were sucked into the nozzles during 3-min pumping. The throughput of cell suction was 1.1 ± 0.2 cells/min (N = 3) for each nozzle. Their distances and speed were measured over time (Figure 7) and the distance of cells from each nozzle were plotted in Figure 7a. Successful cell suction took place within a distance of 150 µm from each nozzle. The other nearby seven cells (two cells on the left, two cells on the middle, and three cells on the right) were attracted to each nozzle, but they did not reach the nozzles and their trajectories were not measured. The 10 cells travelled an average distance of 75.5 ± 26.2 μm (N = 10) and the average aspiration speed was 0.69 ± 0.19 μm/s (N = 10). A cell denoted as #4 in Figure 6 and Figure 7 showed the longest travel distance of 151 µm. The speed of cells increased over time (Figure 7b). The cell speed was around 0.5 μm/s in the initial position and around 1 μm/s in the adjacent nozzles. For more rapid cell manipulation, the manipulation speed needs to be increased. One solution is to increase the number of electrode pairs from seven pairs. 

The cells were transported into the flow channels outside the nozzles. The shut-down of the voltage application stopped the movement of the cells. Three of the 10 cells were flowed through the channels at 3.39 ± 1.65 μm/s (N = 3). The transporting speed of the cells in the channel was much slower than the average flow speed obtained from the previous section. This is because the flow resistance of a micro-nozzle is two orders of magnitudes higher than that of a punched hole. Fluidic resistance through a circular pipe, *R*, is expressed as:

$$R=\frac{128}{\pi }\eta L\frac{1}{D^{4}}$$

where *η* is the dynamic viscosity of the liquid, *L* is the channel length, and *D* is the circle diameter [45]. The ratio of hydraulic resistances of a nozzle and hole can be written as $R_{nozzle}/R_{hole}=L_{nozzle}/L_{hole}{\left(D_{hole}/D_{nozzle}\right)}^{4}$. Inserting *D_nozzle_* = 35 µm, *L_nozzle_* = 70 µm*, D_hole_* = 0.5 mm, and *L_hole_* = 8 mm into the equation yields *R_nozzle_/R_hole_* = 364.

There is potential risk for cell damage associated with electrical field and mechanical stress. When electric field strength is above the electroporation threshold (0.4–0.6 kV/cm), electroporation occurs in the cell and the cell membrane becomes permeable [46,47,48]. In this study, however, the voltage was applied only to the electrodes in the channel, which was far from nozzles. Cells were manipulated without reaching electrodes and their motions were stopped by turning off the voltage. Cells did not need to pass through a high electric field near a pair of electrodes. Therefore, electrokinetic forces on cells can be ignored. Suspended cells were not largely deformed during manipulation and experienced mainly Stokes’ drag in a low Reynolds regime [49]. Since cell speeds were low, we thought strong mechanical stress due to Stokes’ drag was not applied to cells during cell manipulation.

Although it is possible to control the flow through a single nozzle electrically, the bottleneck in large-scale single cell manipulation is the number of signal generators. Analog demultiplexer [50] enables a decrease in the number of signal generating circuits and to maintain the controllability of multiple channels. The placement of trapped single cells in microwells can be achieved with mechanical stages and a microscope [21,30].

## 4. Conclusions

In this study, 4 × 4 micronozzles were integrated with microchannels and electrodes for parallel manipulation of single cells. A DC-biased AC signal was applied to the interdigital tooth electrode made of ITO or Pt/Ti, and an electrokinetic flow occurred at both electrodes in a low conductivity isotonic buffer. By switching the DC bias voltages from positive to negative, the direction of flow was controlled. Since the electrokinetic flow was not significant at small DC bias on ITO electrodes, there was a threshold value of the voltage for generating the flow. Using a micro-nozzle enabled the generation of flow inward and outward of the micro-nozzle and cell manipulation. A flow generated with the electrokinetic pump transported cells into the micro-nozzle array and it was possible to demonstrate manipulation of single cells using the DC-biased ACEK flow. 

The developed method of integrating nozzles and manipulation method is fundamental for creating high-throughput single manipulation tools accessible to an open-top chip. Our integrated system collected single cells at 1 event/min, whereas an on-demand droplet collection system dispensed single cells at a throughput of 20 events/min [29]. The improvement of the throughput and flow rate required an increase in the number of interdigital-tooth electrodes. The true value of this study is that this integration technology provides individual flow control of each micro-channel and scalability with one-side-open multi-channel devices [29,30]. Our future works includes a viability assay and rearrangement of single cells with the nozzles integrated with pumps. 

## Figures and Tables

**Table 1:** Dimensions of the micro-nozzles integrated with indium tin oxide (ITO) micropumps and electrodes.

Category	Parts	Dimension
Flow channel	Height, *H*	71.9 ± 1.41 μm (N = 16)
	Width of flow channel in electrode section, *B*	189.1 ± 4.57 μm (N = 10)
	Width of the narrower flow channel, *A*	62.0 ± 2.30 μm (N = 16)
ITO electrodes	Width, *C*	65.7 ± 0.91 μm (N = 5)
	Gap between a pair of electrodes, *g*	23.3 ± 1.81 μm (N = 5)
	Pitch, *p*	191.0 ± 1.11 μm (N = 5).
Pt/Ti electrodes	Width, *C*	67.8 ± 0.91 μm (N = 5)
	Gap between a pair of electrodes, *g*	24.5 ± 1.44 μm (N = 5)
	Pitch, *p*	192.7 ± 1.12 μm (N = 5)

## Supplementary Materials

The following are available online at https://www.mdpi.com/2072-666X/11/4/442/s1. Movie S1, S2: Movement of particles (φ2 μm) in a low conductive isotonic buffer at V_pp_ 10 V, 100 kHz. Movie S1: V_DC_ + 3 V and Movie S2: −3 V. Voltage is not applied for 5 s from the start of the movie. Movie S3: Vortex flow near a pair of ITO electrodes, and creation of net flow through a PDMS microchannel. Movie S4: Suction of 10 cells to three micro-nozzles integrated with ITO pumps at V_DC_ −4 V, V_pp_ 10 V, and 100 kHz.
